# In pursuit of an HIV vaccine: an interview with Andrew McMichael

**DOI:** 10.1186/1741-7007-11-60

**Published:** 2013-05-21

**Authors:** Andrew J McMichael

**Affiliations:** 1 Weatherall Institute of Molecular Medicine, University of Oxford, Oxford, England OX3 9DS, UK

## 

Andrew McMichael qualified in Medicine before doing a PhD in Immunology with Ita Askonas and Alan Williamson in the 1970s. His research during this time and later work done in his group has made a major contribution to our understanding of T-cell-mediated immunity against viral infections. Initially he worked on the immune response to influenza, but latterly studying the T cell response against HIV has been a major focus, and his group has designed and tested two candidate HIV vaccines in phase I clinical trials. Based through most of his research career in Oxford, he was knighted in 2008 for services to medical sciences, and has just completed 12 years as Director of the Weatherall Institute of Molecular Medicine.

In this 30^th^ year since the discovery of HIV as the cause of AIDS, we asked Sir Andrew to give us his personal perspective on the progress towards a vaccine.

**Figure:**
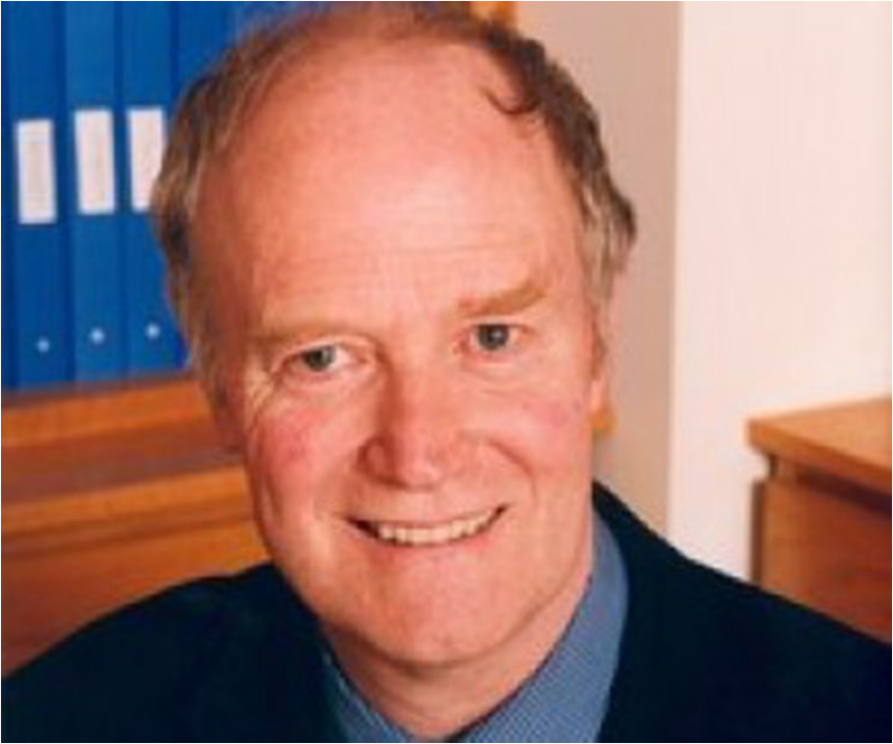
Andrew J McMichael.

## Would you like to start by telling us how and when you first started working on HIV?

We started in 1986 or 1987 when my laboratory was focused on studying immune responses to influenza. I actually had a clinical training fellow - Douglas Nixon - who was studying to be a clinical virologist. He wanted to come and do a PhD and we discussed the possibility of looking at another virus and thought HIV would be worth having a look at. We did some preliminary experiments and found that we could look at T cell immune responses, so we started to work on that. This is all around the time that Alain Townsend’s work on the peptides was getting to a very exciting stage.

## Is this when you and Alain Townsend were discovering how peptides derived from viral proteins were delivered to the surface of infected cells and presented to T cells?

Yes, so we were quite busy. We started working on HIV because at that time there were not many people working on immune responses to HIV. A lot of virologists were already working on it of course. So we thought we’d start on it in quite a small way, but realizing that if the experiments worked, it would probably take over our lives. Which it more or less has done.

Was the hope always to find anti-HIV responses that can protect against infection, and develop a vaccine capable of eliciting these? Why do you think it’s been so difficult?

Well, the vaccine is problematical. But it’s not alone in that. Vaccines for malaria and TB and hepatitis C virus are all difficult problems. So not every vaccine is easy. I think that the reason that HIV is so difficult is that there’s really no example of humans who have become infected, have cleared the virus and are protected from re-infection. So you don’t have that precedent to work with which you have in almost every other virus infection. Then the virus of course is incredibly variable and in fact the more that we know about it, the more variable it appears. That makes it very easy for it to evade immune responses. Also it persists and it can hide as a latent pro-virus. So once infection’s started, it’s very difficult to clear the infection. So for a vaccine to work, you’ve got a very narrow window, probably really just preventing the initial infection. And that’s proving very hard to do with vaccines that primarily elicit antibodies.

## If you think about vaccines that have been successful, have they all been vaccines that induce protective, neutralizing antibodies?

Yes probably, nearly all of them. One possible exception is the BCG vaccine against TB. But BCG is only very partially effective really. All of the really successful - 90% efficient or more - vaccines are vaccines that induce neutralizing antibodies.

## Can you explain, just briefly, why HIV doesn’t seem to be very good at inducing neutralizing antibodies?

It was thought that it was very bad at inducing neutralizing antibodies, although recently there are lots of studies looking at patients who are chronically infected, who do actually make pretty good neutralizing - broadly neutralizing - antibodies, but it’s too late for them. They can take 3 to 5 years before they come up. One of the problems is that the first antibodies that are made are not neutralizing. Within two weeks of infection people will make some antibodies to parts of the virus - the envelope protein - but they are non-neutralizing. And then there’s a rather long period before they make antibodies that will neutralize the autologous or infecting virus - that’s about 80 days. Then it may be years before they make antibodies that broadly neutralize many different viruses. The reasons, I think, are partly that there are many sites where the antibody seems to bind to but doesn’t effectively neutralize. There are many sites where the virus just varies - where it has variable loops - so that the antibodies might neutralize that virus, but it can very easily escape by mutation. And then there are relatively few sites that are highly conserved, which the virus can’t really change without big fitness costs, such as the CD4 binding site which it uses to infect cells [1]. It seems to be very hard to get antibodies to those sites. They do come up in some patients after 3 to 5 years, but these antibodies are very mutated from their germline ancestor antibodies. There’s been a lot of selection over several years. So sometimes they accumulate 50 somatic mutations before they acquire the specificity and affinity of a broadly neutralizing antibody. But even then they are a minority population in all the antibodies - they’re mainly present at under 1% of all the antibodies being made. The challenge is to design a vaccine that will somehow induce lots of these protective antibodies [2]

## What about T cell immunity - is there evidence of that being effective in controlling the infection?

There’s good evidence that cytotoxic CD8 T cells will control or partially control the infection once it’s started and that indeed good T cell responses will control better than poor T cell responses. They may have to be good in a number of senses - to be good killers, to make good cytokines and chemokines, to target, again, the more conserved regions of the virus so that the virus can’t escape very easily and to be present in adequate numbers to control the response. Because CD8 T cells detect intracellular infections when the viral peptides are presented at the cell surfaces by host HLA molecules, which are highly polymorphic, you get particular HLA types being favorable, such as HLAB57, which, probably by chance, picks out conserved regions of the Gag protein. It may also be that T cell responses targeted to Gag are more effective than T cell responses to Envelope - there’s good evidence for that. Not clear why, although Gag is more conserved. So a number of factors give variable T cell responses and some people control better than others. But the T cell responses are important in getting some degree of control of infection [3].

## And is this the basis on which you’ve been working towards designing a T cell vaccine?

Well, we’ve been arguing that there’s a good case for a T cell vaccine as a backup to an antibody-inducing vaccine or as a possible alternative, at least in those who were not protected by the antibody vaccine. If you couldn’t get 100% efficacy, then having a strong T cell response may be useful as it would lead to better control of the virus. Indeed, there are many infections we have, such as Epstein-Barr virus and cytomegalovirus, where the T cells permanently control the infection within people, and they remain healthy. So you could envisage a situation where we have strong T cell responses to HIV and HIV controlled long term and at very low levels, where it’s not causing any trouble. Rather like being on permanent anti-retroviral therapy. There’s also a study in macaques from Louis Picker in the last two or three years which shows that if he can establish very strong early T cell responses, that he could, in some cases, eradicate the infection in the monkeys via T cells [4].

## So in that case, a T cell vaccine really worked?

Yes, the macaques were given a rather special vaccine which was vectored by cytomegalovirus, which is a persistent infection, which I’ve just said is usually not terribly harmful on its own but maintains a very strong CD8 T cell response to cytomegalovirus, in this case with inserted HIV antigen. This seems to be particularly effective at stimulating and maintaining effective T cell responses, and in some cases eradicating the virus when it is subsequently given as a challenge.

## Is this a vaccine vector that could be used in humans?

There’s an argument about that because it would be a persisting infection. So once given, it would be difficult to get rid of it if it was found to have side effects 20 years later. Although it’s thought to be a fairly harmless virus, there are situations where it can cause disease, particularly in immunodeficiency. A disease caused by the vaccine would not be a very good situation. So there are some concerns that it might not be quite as safe as people who are advocating it sometimes suggest. I think the regulators would have concerns. The alternative is to try and find something else that would be safer, that would stimulate the same kind of T cell response.

## Could you briefly explain the design of the particular vaccines that you are developing?

We’re trying to get the immune response to focus on conserved viral peptides that can be presented to T cells. So we’re making an artificial construct, which incorporates fragments of the HIV genome that are fairly well conserved across the major clades. In our initial analysis, we chose regions that were less than 6% variable in amino acid sequence across the four major clades. Then we stitched those 14 fragments together so it’s about a quarter of the total of HIV. They are then used as an immunogen. Working with Tom Hanke, who designed it with me [5], and Lucy Dorrell, who does all the clinical studies, we’ve done some phase 1 trials with it [6]. We’re getting quite encouraging results. At least we can get quite strong immune responses to this candidate vaccine.

Is there quite a lot of variability in how strong the responses are across the population, given that everybody has different antigen presenting HLA molecules? Is that a difficulty?

In phase 1 trials we’ve tested it in about 30 people so far - they all respond to it, to varying degrees and varying breadths. Some will respond to multiple peptides, others probably to fewer peptides. As we dissect that, that may turn out to be important. We may have to figure out ways to try and ensure that everybody, whatever their HLA type, has a certain level of breadth in their response.

## If you look at the people who do control HIV well, do they have a good breadth of response to different conserved viral peptides?

We’ve just published a paper describing this [7], although it was a mix of people, some with good HLA types and some without. We were looking at the rate at which the virus escaped from the T cell responses in acute infection. The more conserved across the viruses that the peptide epitope seen by T cells is, the slower the virus would escape from this T cell response by a mutation. The other factor that was important was that if the response to that peptide was strongly immunodominant, the virus would escape more rapidly. If you put together an immunodominant response and a slow escaping conserved epitope, then you must get the strongest pressure on the virus. People with protective HLA types do exactly that. They make strongly immunodominant responses to conserved epitopes. So we think that what we need to do with a vaccine is to get these strong responses to conserved epitopes, maybe sometimes epitopes that in people with less favorable HLA types would not normally respond to. But if a vaccine could stimulate those strong dominant responses, they may get much better protection.

## Presumably if the virus does manage to escape an immunodominant response to a conserved epitope, it will be at some fitness cost?

Well that’s the hope. There is some evidence that that is the case for some of these well studied epitopes. But the idea would be to make that a sort of general situation so that if the virus did escape, it will be less fit. And then you maintain some advantage over the virus.

## Are you getting to the point where you’d be able to progress from phase 1 trials onto phase 2?

Well, we’d like to. What we’re doing at the minute is we’re dissecting these immune responses in as much detail as we can. We’re particularly interested to see how well these T cells suppress virus-infected cells, not just looking at the T cell effector functions, like killing or cytokine production, but actually looking at their ability to suppress autologous CD4 cells infected with a variety of viruses to see whether we get something that’s equivalent to the holy grail for the neutralizing antibodies - neutralizing a whole variety of different viruses *in vitro*. We can do the same kind of thing with the T cells, suppressing their replication *in vitro*. Then we think we could sort of predict whether this vaccine might work or have an effect *in vivo*. The more we can do *in vitro*, the cheaper and easier and faster it is. And we can gradually build a case to try and test this in an efficacy trial *in vivo*.

## Are there any animal models you can use?

Well, it’s a little tricky. You could do some pllel experiments in monkeys for SIV, but it wouldn’t actually be the same vaccine because of the variation within SIV and HIV is not identical. We’d have to redesign a whole new construct, so it wouldn’t be an exact pllel. But we have discussed that and we are thinking about it.

## Are humanized mice any use for testing vaccines of this type?

The problem is, I think, they don’t make terribly strong T cell responses. They do make some, but they’re quite difficult to work with I think. And obviously expensive and labor intensive. But they could be useful and that’s something we probably need to look at again. My feeling would be, if we could get this vaccine stimulating really strong virus inhibition across a range of viruses by CD8 T cells *in vitro*, then we could make quite a strong case for it to be tested *in vivo* for efficacy. But I think we wouldn’t be in a position to argue that for a year or two yet. Then to go into an efficacy trial, it’s a huge hurdle of getting someone to fund it because they cost millions and they take years and they take huge teams. I think it would have to be tested alongside a pllel construct that would generate and accumulate neutralizing antibodies.

## And then how would you know which was the most effective?

I think that if you got no infections, that would be the antibody. That would be the wonderful result, that you had complete protection. On the other hand, if you did get some infection, then you’d look at the degree of control of those infections, relative to the placebo group. And you would be looking to see whether you got lower virus loads and slower progression. You could probably tell which was doing which.

## If you look at the recent Thai vaccine trials that reported a modest efficacy, did they see any reduction in viral load?

No, they didn’t really have any CD8 T cells to speak of [8]. But it would be that sort of result. If you got a result where maybe you got 50% efficacy in terms of full protection, which would be very good, and then in the other 50% that got infected, you’d be looking to see whether the CD8 T cells had given you lower viral loads. You could then talk about how long could they be safe going before they’d have to go on medication. Ideally, you’d want that number to be decades.

## Well that would be a tremendous advance

It would. I don’t think it’s out of reach. It’s difficult. And it may be someone else’s vaccine will do it better than ours. But I think that is an achievable aim.

## Is your vaccine designed to target the mucosal part of the immune system?

Well, I’m a bit more lukewarm about that. With the sort of T cell vaccine we’re thinking of, we’re probably coming in to act after the initial mucosal infection has spread into lymphoid tissues. You could say that the macaque vaccine from Louis Picker is similar, in that it’s a T cell vaccine, and it doesn’t eradicate the infection within the first 5 or 7 days when it’s in the genital mucosa or rectal mucosa. It’s curing the infection perhaps weeks, or months, later when it’s already spread around the body.

## So that result is possibly the most encouraging one yet?

Well it’s very exciting. But there’s also lots of things puzzling about it. It doesn’t happen in all macaques. So why doesn’t it happen in some of them? What are they doing differently? I’m not clear if we know what the answer to that is. But again, it’s something that should be solvable.

## Would you say that your vaccines are generating T cell immunity in a way that is different from other vaccines such as the STEP vaccine?

The STEP vaccine trial in 2009 tested whether a vaccine (adenovirus-5 vectored HIV Gag Pol and Nef) could enable better control of virus in those infected, but it did not [9]. I think our vaccine is doing something a bit different by focusing T cell responses only on the conserved regions of the virus so that vaccine-virus matches are better and escape is less likely. I know there are some other similar approaches, at various stages of development, that may be doing similar things now that are not published. We’re trying to do something different and it looks like we’re sort of part of the way there. Although I think we still need to do a bit more to be sure it’s going to work. Or has a chance of working.

## It sounds like you’re an optimist

Well you have to be an optimist to work in this field.

I’d like to add, just briefly, that there’s a lot of interest in trying to eradicate the virus in patients with mixtures of drugs and activators of latent virus. Adding in an immune response, as well, might help that process. So these sorts of combined approaches could be used in therapeutic strategies. One of my colleagues, Lucy Dorrell, is working on that.

That sounds promising. Has this approach been tried with other viral vaccines to persistent viruses, such as the vaccine against human papillomavirus? Has that been tested in people who already have the virus?

Yes there’s quite a bit of work going on there. I’m not sure it can eradicate the infection - that’s the trouble. But again, that’s a vaccine aimed at stimulating antibodies. You may need more cellular immune responses to eradicate. I think it’s an interesting idea that one might, for some of these persisting viruses, not just HIV, but hepatitis C, for example, be able to use combinations of drugs and immune responses in an aggressive sort of manner to eradicate the virus and then, essentially, cure people. There’s certainly a lot of interest in that.

Will it actually be a lot easier to get a trial going to test that idea, in the case of HIV and your vaccines, than to do the trials to establish whether there’s protection?

It would be easier than HIV vaccine efficacy trials, which really do cost millions and millions. An eradication type of study, one could do on a small group of patients. Because at the moment if you can do it in one patient, you’d cause a sensation. If you can do it in one, you can probably do it in more.

## Note

This article is part of the cross journal collection *HIV thirty years on*. Other articles in this series can be found at [10].
